# Molecular profiles of small cell lung cancer subtypes: therapeutic implications

**DOI:** 10.1016/j.omto.2021.02.004

**Published:** 2021-02-06

**Authors:** Anna Schwendenwein, Zsolt Megyesfalvi, Nandor Barany, Zsuzsanna Valko, Edina Bugyik, Christian Lang, Bence Ferencz, Sandor Paku, Andras Lantos, Janos Fillinger, Melinda Rezeli, Gyorgy Marko-Varga, Krisztina Bogos, Gabriella Galffy, Ferenc Renyi-Vamos, Mir Alireza Hoda, Walter Klepetko, Konrad Hoetzenecker, Viktoria Laszlo, Balazs Dome

**Affiliations:** 1 Department of Thoracic Surgery, Comprehensive Cancer Center Vienna, Medical University of Vienna, 1090 Vienna, Austria; 2 Department of Thoracic Surgery, National Institute of Oncology-Semmelweis University, 1122 Budapest, Hungary; 3 National Koranyi Institute of Pulmonology, 1121 Budapest, Hungary; 4 1st Department of Pathology and Experimental Cancer Research, Semmelweis University, 1085 Budapest, Hungary; 5 Department of Biomedical Engineering, Lund University, 221 00 Lund, Sweden; 6 Torokbalint County Institute of Pulmonology, 2045 Torokbalint, Hungary

**Keywords:** small cell lung cancer, heterogeneity, neuroendocrine, molecular profile

## Abstract

Small cell lung cancer (SCLC; accounting for approximately 13%–15% of all lung cancers) is an exceptionally lethal malignancy characterized by rapid doubling time and high propensity to metastasize. In contrast to the increasingly personalized therapies in other types of lung cancer, SCLC is still regarded as a homogeneous disease and the prognosis of SCLC patients remains poor. Recently, however, substantial progress has been made in our understanding of SCLC biology. Advances in genomics and development of new preclinical models have facilitated insights into the intratumoral heterogeneity and specific genetic alterations of this disease. This worldwide resurgence of studies on SCLC has ultimately led to the development of novel subtype-specific classifications primarily based on the neuroendocrine features and distinct molecular profiles of SCLC. Importantly, these biologically distinct subtypes might define unique therapeutic vulnerabilities. Herein, we summarize the current knowledge on the molecular profiles of SCLC subtypes with a focus on their potential clinical implications.

## Graphical Abstract

**Figure:**
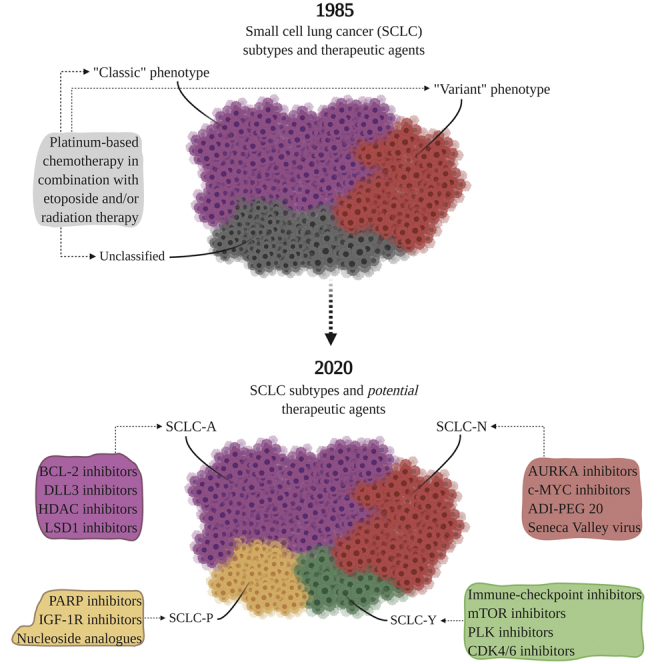

## Teaser

Small cell lung cancer is still regarded as a homogeneous disease associated with poor prognosis. Recent analysis, however, has led to the development of novel subtype-specific classifications primarily based on the neuroendocrine features and molecular profiles. The better understanding of these biologically distinct subtypes might help to define unique therapeutic vulnerabilities.

## Main text

Lung cancer, the leading cause of cancer-related deaths in the Western world, is classified into two major groups: small cell lung cancer (SCLC) and non-SCLC (NSCLC).^1^ SCLC accounts for approximately 13%–15% of all lung cancers, and with a 5-year survival rate of less than 7%, it remains one of the most lethal forms of malignant diseases.^2^^,^^3^ It has a very aggressive course and is characterized by genomic instability, almost universal inactivation of the genes *TP53* and *RB1*, rapid tumor growth, increased vascularity, and high metastatic potential.^4^, ^5^, ^6^ Consequently, at the time of diagnosis, most SCLC patients already present with a metastatic spread outside the chest, which often leads to premature death.^7^^,^^8^ Most SCLC patients are current or former heavy smokers resulting in a high tumor mutational burden (TMB) (with C:G>A:T transversions being the most common type of base substitutions).^9^^,^^10^ Early detection strategies are mostly ineffective for SCLC even among high-risk populations, and there have been no significant improvements in survival and therapeutic approaches for more than 30 years, leading SCLC to be categorized as a “recalcitrant” cancer.^5^^,^^11^

**Figure 1:**
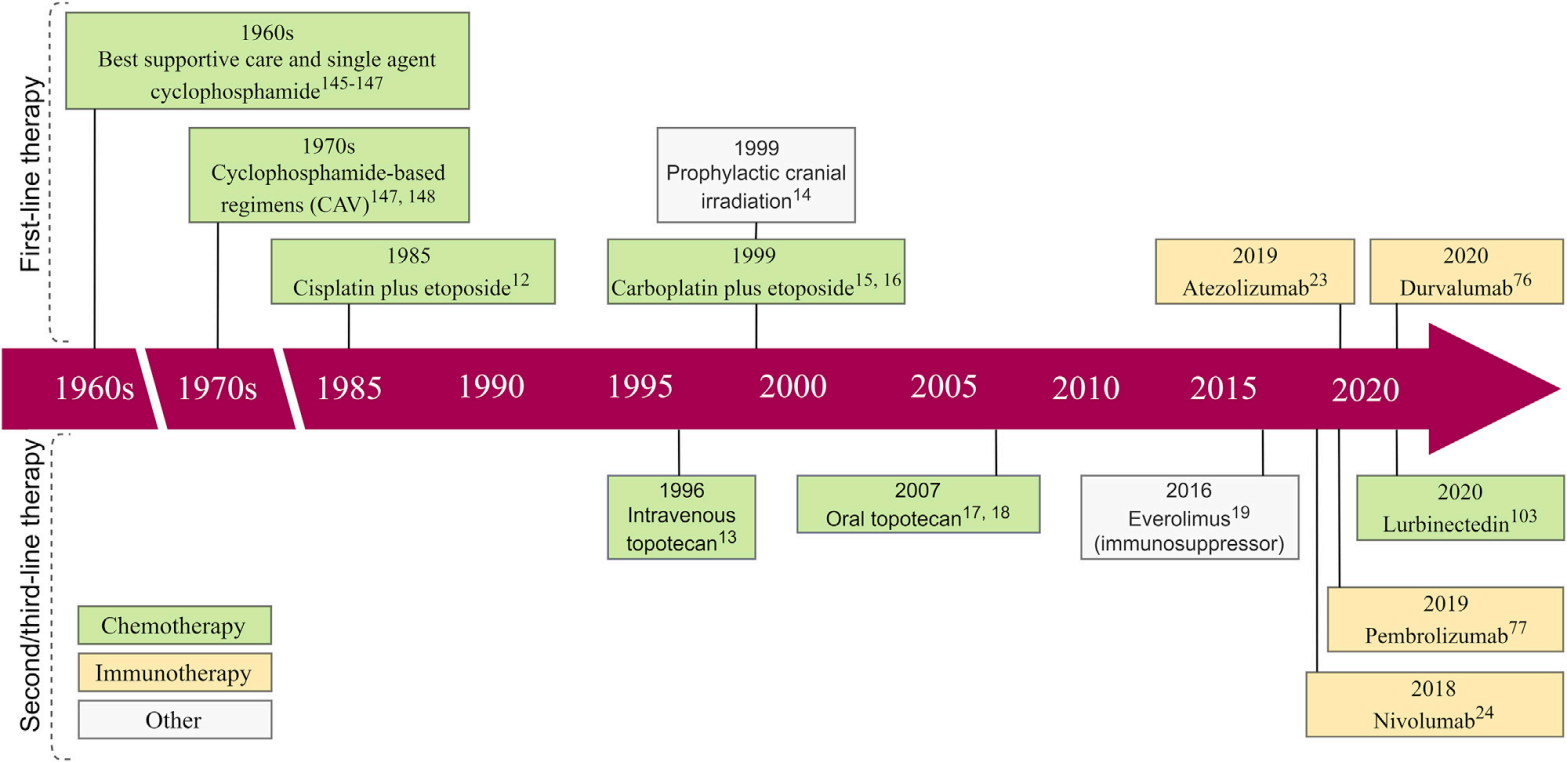
Timeline of relevant therapeutic advances for small cell lung cancer (SCLC) Initial therapeutic strategies for SCLC included surgery or radiotherapy alone. However, given the aggressive behavior and high metastatic potential of SCLC, systemic therapy with cytotoxic agents has rapidly become the cornerstone of management. In the 1940s, alkylating agents (such as nitrogen mustard) were used for the treatment of all bronchogenic carcinomas (including SCLC), resulting in tumor regression in more than 50% of patients. Yet, at that time, the true nature of SCLC was widely unknown, and all lung carcinomas were treated similarly. The first chemotherapeutic agent to show a statistically significant survival benefit for selected SCLC patients was cyclophosphamide, which doubled the survival when compared to best supportive care alone 12, 13, 14. Despite the encouraging results with single-agent therapy, it became obvious early in the 1970s that combination therapy produces superior survival outcomes when compared to single-agent treatment 14, 15. Therefore, during the late 1970s and early 1980s, cyclophosphamide was used in combination with other cytotoxic agents such as doxorubicin and vincristine (CAV). The basis of the currently used platinum-based combination chemotherapy (CHT) was defined during the mid-1980s when Evans et al.^16^ showed a clear survival benefit for patients treated with cisplatin plus etoposide. Since then, there have been no relevant advances in the standard-of-care CHT regimens, and the backbone for current combination strategies are still the platinum compounds. In more recent years, targeted therapy and immunotherapy have also been actively tested, leading to the approval of several immune-checkpoint inhibitors such as atezolizumab, pembrolizumab, and nivolumab.

Platinum-based chemotherapy (CHT) in combination with etoposide and/or radiation therapy (RT) has been used in SCLC treatment and still remains the backbone for current combination strategies (Figure 1^17^, ^18^, ^19^, ^20^, ^21^, ^22^, ^23^).^16^^,^^24^ Unlike NSCLC, which has an intrinsic tendency for CHT resistance, SCLC is initially highly sensitive to cytotoxic agents.^25^ Even with response, however, SCLC frequently recurs within a short time span, and patients are seldom cured.^26^ The most notable recent clinical progress in SCLC was the approval of the immune-checkpoint inhibitors (ICIs) atezolizumab, pembrolizumab, and nivolumab.^27^^,^^28^ Unfortunately, however, only 12.6% of patients remain progression-free at 1 year, and to date there are no reliable biomarkers predicting response to immune checkpoint blockade.^27^^,^^29^ With regard to other therapeutic approaches, due to the rapid doubling time of tumors and their high propensity to metastasize, surgery is rarely performed.^30^ Accordingly, limited human SCLC tissue availability has increased the importance of preclinical models.^5^^,^^31^

Unlike the increasingly personalized approach to clinical care of patients with other types of lung cancer, SCLC is still regarded as a “homogeneous” disease with a single morphological type.^32^ Consequently, current clinical study protocols for SCLC are generally based on disease stage with no consideration of predefining distinct molecular marker expressions that might have predictive or prognostic significance.^5^^,^^33^ However, recently, there has been a worldwide resurgence of studies on SCLC, including the development of new preclinical models (such as patient-derived xenografts [PDXs] and genetically engineered mouse models [GEMMs]), comprehensive genomic profiling, and the identification of biologically distinct molecular subtypes.^34^ Exploring the molecular profiles of SCLC subtypes might help to focus and accelerate therapeutic research. In this review, we systematically analyze the molecular heterogeneity of SCLC, mainly focusing on rationally targeted therapeutic implications and new treatment opportunities that may ultimately improve the clinical outcomes for patients with this devastating disease.

### Tumor heterogeneity in SCLC

#### Neuroendocrine (NE) subtypes

**Figure 2:**
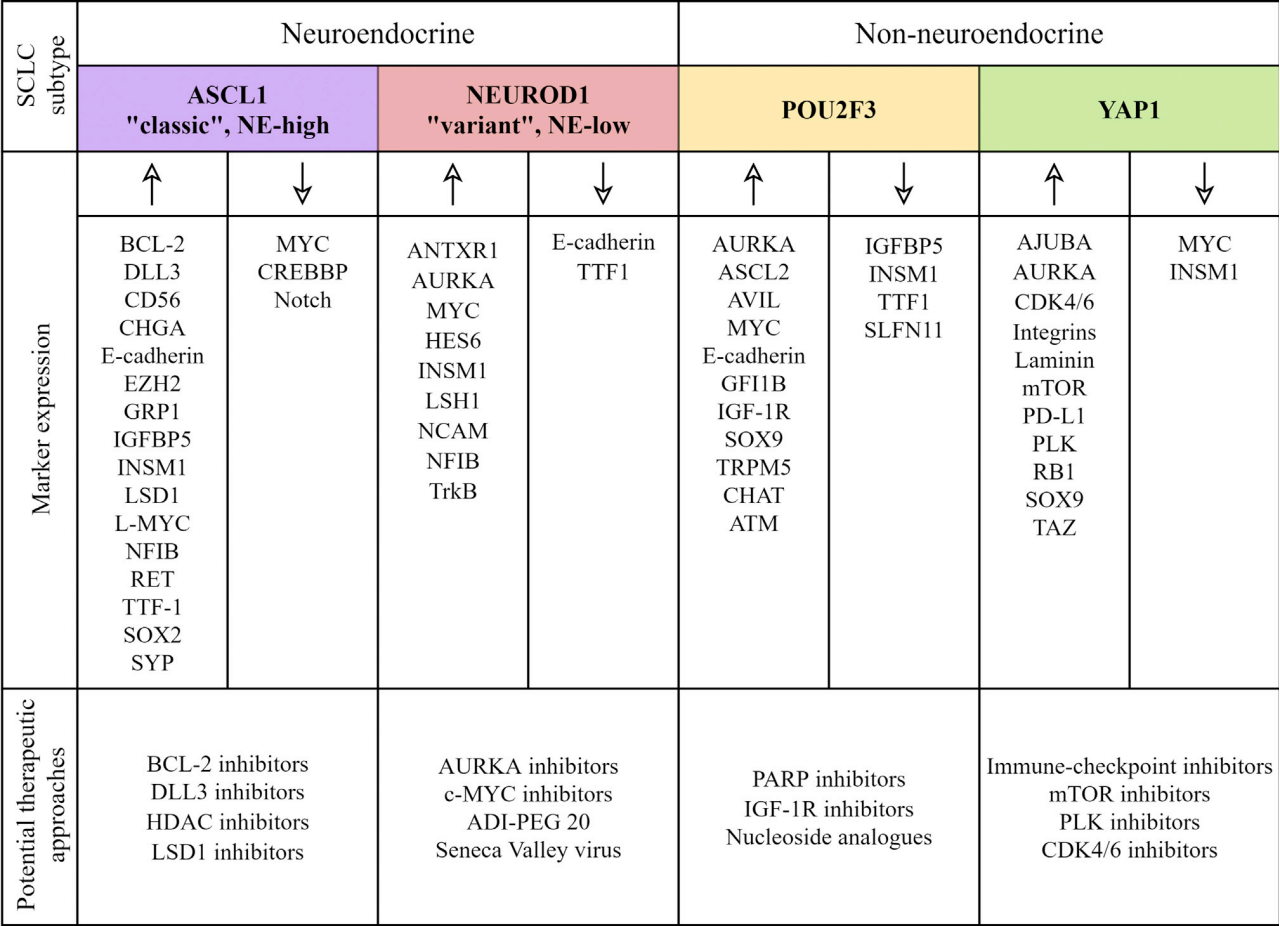
Tumor heterogeneity in SCLC with regard to neuroendocrine differentiation, molecular subtypes, and gene expression profile Neuroendocrine (NE) differentiation can be defined by the expression pattern of different NE markers, including chromogranin A, synaptophysin, neural cell adhesion molecule 1, and gastrin-releasing peptide. However, a minority of SCLCs are negative for all standard NE markers. Additionally, SCLC can be subclassified according to the relative expression of four key transcriptional regulators: achaete-scute homolog 1 (ASCL1; also known as ASH1), neurogenic differentiation factor 1 (NEUROD1), yes-associated protein 1 (YAP1), and POU class 2 homeobox 3 (POU2F3). Various genetic alterations, biological properties, and thus potential therapeutic vulnerabilities are associated with these molecular subsets.

Although clinically SCLC is still regarded as a single disease entity, preclinical studies from the past decades identified biologically different SCLC subgroups (Figure 2). Accordingly, SCLC can be classified today into NE-high and NE-low subtypes primarily based on the expression pattern of different NE markers such as chromogranin A (CHGA), synaptophysin (SYP), neural cell adhesion molecule 1 (NCAM1/CD56), and gastrin-releasing peptide (GRP).^5^^,^^6^^,^^33^^,^^35^ Additionally, some SCLCs lack NE differentiation and are termed as non-NE tumors.^33^ The NE-high versus NE-low subtypes show major differences in genetic alterations, morphology, growth properties, and immune infiltration.^35^ NE-low SCLCs are furthermore associated with increased immune cell infiltration and referred to as “immune oasis” tumors, whereas NE-high SCLCs are characterized by low numbers of infiltrating immune cells and, consequently, have an “immune desert” phenotype.^36^ This categorization has major clinical impact, as the two phenotypes are anticipated to respond differently to targeted therapeutics and ICIs.^37^^,^^38^

Of note, another example of NE heterogeneity was described by Gazdar et al.^39^^,^^40^ in 1985 based on the *in vitro* and *in vivo* behavior of SCLC cells. The “classic” phenotype is associated with typical morphology, high expression of NE markers, and non-adherent growth pattern in cell cultures.^5^^,^^39^ In contrast, the “variant” phenotype is usually characterized by larger cells with prominent nucleoli, low expression of NE features, and an adherent or loosely adherent growth pattern *in vitro*.^5^^,^^33^^,^^35^

#### Molecular subtypes

Recent SCLC profiling studies on cancer cell lines, PDXs, GEMMs, and primary human tumors suggest a model of distinct subtypes defined by the relative expression of four key transcriptional regulators: achaete-scute homolog 1 (ASCL1; also known as ASH1), neurogenic differentiation factor 1 (NEUROD1), yes-associated protein 1 (YAP1), and POU class 2 homeobox 3 (POU2F3).^33^^,^^41^ The association between these transcription factors (TFs) and the NE expression profile may provide subtype-specific therapeutic vulnerabilities (Figure 2).^33^ SCLC-A (ASCL1) tumors show high expression of NE markers and classic morphology, compared to the NE-low SCLC-N (NEUROD1) subtype with variant morphology.^34^ The transcription regulators ASCL1 and NEUROD1 both have been implicated as essential determinants of the developmental maturation of pulmonary NE cells (PNECs).^42^ In addition, SCLC-A and SCLC-N subtypes preferentially express the TF insulinoma-associated protein 1 (INSM1) as well.^33^ By inhibiting the Notch signaling pathway, INSM1 also plays a key role in NE differentiation, and thus its expression levels are low in non-NE SCLCs.^43^, ^44^, ^45^ NEUROD1, ASCL1, and INSM1 low-expressing or non-expressing SCLCs are classified either into SCLC-Y (YAP1) or SCLC-P (POU2F3) subtype, depending on their TF expression pattern.^33^ YAP1 is a transcription regulator activated by the HIPPO signaling pathway.^45^ Meanwhile, POU2F3 is required for the development of pulmonary tuft cells and chemosensory cells of the gastrointestinal epithelium.^46^ Accordingly, recent data on high POU2F3 expression patterns detected in certain SCLCs suggest tuft cells as the origin of the SCLC-P subtype.^33^^,^^46^

#### Genomic landscape of SCLC

SCLCs are likely to gain various genetic alterations as they evolve or as they metastasize outside the chest.^47^ The metastatic potential of SCLC is often driven by the overexpression of nuclear factor I B (NFIB), which functions as an oncogene and is frequently amplified in metastases.^48^ Likewise, lymph node (LN) metastases often undergo a change in NE expression patterns compared to the primary cancer, thus enhancing the aggressive course of the disease.^6^^,^^49^ It is important to consider intratumoral heterogeneity in SCLC, as NE cancer cells are also capable of raising non-NE tumor cells that expedite the tumorigenesis and additionally contribute to CHT resistance.^47^ Meanwhile, cancer stem cells (CSCs) are suspected to promote long-term tumor growth and affect the cellular heterogeneity of SCLC.^47^ In this context, SOX2, the transcriptional regulator of pluripotent stem cells, is frequently overexpressed, especially in the SCLC-A subtype.^50^

Notch signaling in SCLC is involved in NE differentiation.^51^ Tumor cells, which exhibit active Notch signaling, are slowly growing and often chemoresistant. Accordingly, Notch is considered as a tumor suppressor in SCLC.^51^^,^^52^ Based on the inhibitory activity of DLL3 on the Notch pathway or the inactivating mutations in Notch pathway genes, Notch is frequently inactivated in SCLCs with a high NE expression profile.^6^ Loss of NE differentiation and concomitant activation of Notch signaling is facilitated by the activation of the RE1 silencing transcription (REST) factor, a transcriptional repressor of NE and neuronal differentiation.^53^ Notably, REST is absent in most NE SCLCs, also resulting in the inhibition of Notch signaling.^5^

NE-high SCLCs often harbor overexpression in one of the Myc family members, including c-MYC, l-MYC, and n-MYC.^54^ The SCLC-A subtype is highly associated with the expression of l-MYC, whereas the upregulation of c-MYC is related to the SCLC-N subtype.^33^ In addition, the SCLC-A subtype is suspected to lose ASCL1 expression as a result of c-MYC overexpression since high levels of c-MYC might contribute to the progression of SCLC cells from the classic phenotype to the NE-low variant phenotype.^55^

It has also been hypothesized that *MYC* amplification appears during tumor progression and is connected to CHT resistance.^54^^,^^56^ Aurora kinase A and B (AURKA and AURKB) are serine/threonine kinases and have a main function in the regulation of mitosis.^57^ The overexpression of AURKA promotes cell proliferation.^58^ Meanwhile, AURKB phosphorylates RB1 and regulates the postmitotic checkpoints as well as prevents polyploidy after irregular mitosis.^55^ The overexpression of aurora kinases in SCLC accompanied by Myc family amplification provides a growth advantage and causes polyploidy in SCLC.^59^

The programmed cell death protein 1 (PD-1)/programmed death-ligand 1 (PD-L1) signaling pathway is a major therapeutic target in SCLC.^60^ A considerable proportion of SCLCs exhibit aberrant PD-L1 expression on tumor cells that may be fundamental for moderate responses to immunotherapy.^61^ A subset of SCLCs is associated with the amplification of the fibroblast-growth factor receptor 1 (*FGFR1*) gene that displays a target for FGFR1 inhibitor therapy.^62^ Other unique features in SCLC include the expression of TFs SOX9 and ASCL2 and the receptor tyrosine kinase insulin-growth factor receptor 1 (IGF-1R) in POU2F3-expressing tumor cells.^46^ Important aberrations in the SCLC-A subtype comprise amplifications of *BCL2*, *EZH2*, and the decrease of *CREBBP*, whereas SCLC-Y is associated with mutations in the phosphatidylinositol 3-kinase (PI3K)/AKT/mTOR signaling pathway.^5^ Conclusively, intertumoral heterogeneity in SCLC has major therapeutic impacts.

### Therapeutic implications

#### CHT

SCLC is initially highly sensitive to CHT and, accordingly, up to 75%–80% of all SCLCs first respond to platinum compounds.^63^ However, the development of resistance is essentially universal, and patients are rarely cured. Chemorefractory tumor cells may arise due to the extensive TMB in SCLC and the coexisting subpopulations within a tumor.^5^^,^^47^^,^^64^. In addition, therapeutic outcomes might also be altered by the growth of resistant cell clones during disease progression.^47^ Recent data suggest increased intratumoral heterogeneity after the onset of therapeutic resistance in SCLC.^34^^,^^65^ In contrast to NE-high SCLC cells (which are more sensitive to CHT because of their fast proliferation and high mitotic rate), the slower growing SCLC cells such as the previously mentioned Notch^active^ SCLC cells may be inherently chemoresistant.^47^ Inactive Notch signaling triggers the NE cell differentiation during lung development, while active Notch signaling controls the non-NE cell fates.^66^ Notch signaling further mediates the transition of NE to non-NE phenotype.^66^ In SCLC, Notch signaling delivers context-dependent tumor-suppressive or oncogenic signals through its receptors.^66^ MYC is also hypothesized to mediate NE plasticity in SCLC by activating Notch signaling.^51^^,^^67^ Furthermore, this interaction controls the dynamic behavior of cancer cells contributing to the co-existence of subtypes within a tumor.^51^^,^^67^

The expression of Schlafen 11 (SLFN11) (a member of the Schlafen family involved in the control of cell proliferation and induction of the immune response) is observed to predict treatment responses to DNA-damaging agents such as cisplatin, etoposide, and poly(ADP-ribose) polymerase (PARP) inhibitors in SCLCs.^34^^,^^68^^,^^69^ SLFN11 expression, therefore, correlates with sensitivity to various DNA-damaging chemotherapeutics, but in many cases it is silenced in SCLC by methylation or acetylation.^34^^,^^70^ On the contrary, the upregulation of EZH2 mediates chemoresistance based on SLFN11 downregulation via histone methylation and modification.^71^ Preclinical data led to the hypothesis that therapeutic targeting of EZH2 might prolong and augment sensitivity to the CHT response.^60^

#### Immunotherapy

ICIs have had a major impact on the clinical outcome of several solid tumors, including NSCLC, melanoma, and urothelial cancer.^34^^,^^64^^,^^72^^,^^73^ Epidemiological, biological, and clinical features of SCLC suggest that immunotherapy might be effective in this malignancy as well since many of the ICI susceptibility features in NSCLC are even more pronounced in SCLC.^33^^,^^34^^,^^74^ First, SCLC occurs almost exclusively in heavy smokers, and exposure to cigarette smoking is a predictive factor for responsiveness to ICIs in NSCLC.^75^^,^^76^ Second, compared to NSCLCs, which exhibit a lower TMB of 6.3–9 mutations/Mb, SCLCs display a higher median TMB of 9.9 mutations/Mb and lack the recurrent driver alterations in *EGFR* or *ALK* that correlate with poor response to immunotherapy.^24^^,^^77^ Third, SCLCs can also spontaneously provoke a strong immune response based on the segregation of humoral or cellular components that are summarized as paraneoplastic syndromes (PNSs).^78^ However, despite the above-mentioned susceptibility features, the latest clinical trials regarding the treatment of SCLC with ICIs have shown only modest improvement both in progression-free survival (PFS) and overall survival (OS) (Table 1).^74^

**Table 1:** Completed clinical trials evaluating the safety and efficacy of immune-checkpoint inhibitors in SCLC patients

Study name	Study phase	Mechanism of action	Agent	Outcomes
**First-line therapy**				

IMpower133^27^	I/III	PD-L1 inhibitor	atezolizumab	safety: well tolerated
				ORR: 60.2% versus 64.4%
				PFS:^a^ 5.2 versus 4.3 (p = 0.02)
				OS:^a^ 12.3 versus 10.3 (p = 0.007)
CASPIAN^80^	III	PD-L1 inhibitor	durvalumab	safety: well tolerated
				ORR: 79% versus 70%
				PFS: 5.1 versus 5.4 (p = ns)
				OS: 13.0 versus 10.3 (p = 0.004)
KEYNOTE-604^138^	III	PD-1 inhibitor	pembrolizumab	safety: well tolerated
				ORR: 70.6% versus 61.8%
				12-month PFS: 13.6% versus 3.1% (p = 0.0023)
				24-month OS: 22.5% versus 11.2% (p = 0.0164)
REACTION^139^	II	PD-1 inhibitor	pembrolizumab	safety: well tolerated
				ORR: 61%
				PFS: 4.7 versus 5.4
				OS: 12.3 versus 10.4
ClinicalTrials.gov: NCT01450761^140^	III	CTLA-4 inhibitor	ipilimumab	safety: well tolerated
				ORR: 62% versus 62%
				PFS: 4.6 versus 4.4 (p = 0.016)
				OS: 11.0 versus 10.9 (p = 0.377)

**Maintenance**				

CheckMate 451^141^	III	PD-1 and CTLA-4 inhibitors	nivolumab plus ipilimumab	non-significant results
ClinicalTrials.gov: NCT02359019^142^	II	PD-1 inhibitor	Pembrolizumab	safety: well tolerated
				ORR: 11.1%
				PFS: 1.4
				OS: 9.6

**Recurrent SCLC**				

CheckMate-032^143^	I/II	PD-1 and CTLA-4 inhibitors	nivolumab/ nivolumab plus ipilimumab^b^	safety: manageable safety profile
				ORR: 10% versus 23% versus 19%
				PFS: 1.4 versus 2.6 versus 1.4
				OS: 4.4 versus 7.7 versus 6.0
BIOLUMA^c^,^144^	II	PD-1 and CTLA-4 inhibitors	nivolumab plus ipilimumab	safety: high toxicity rates
				ORR: 38.8%
CheckMate-331^145^	III	PD-1 inhibitor	nivolumab	safety: well tolerated
				ORR: 14% versus 16%
				PFS: 1.4 versus 3.8
				OS: 7.5 versus 8.4
KEYNOTE-028^146^	Ib	PD-1 inhibitor	pembrolizumab	safety: well tolerated
				ORR: 33.3%
				PFS: 1.9
				OS: 9.7
KEYNOTE-158^147^	II	PD-1 inhibitor	pembrolizumab	safety: well tolerated
				ORR: 18.7%
				PFS: 2.0
				OS: 9.1
ClinicalTrials.gov: NCT02261220^148^	I/II	PD-L1 and CTLA-4 inhibitors	durvalumab plus tremelimumab	safety: well tolerated
				ORR: 13.3%
				PFS: 1.8
				OS: 7.9

PD-1, programmed cell death protein 1; PD-L1, programmed death-ligand 1; CTLA-4, cytotoxic T lymphocyte-associated protein 4; ORR, objective response rate; PFS, progression-free survival; OS, overall survival.

a In months.

b Nivolumab plus ipilimumab combination consisted of nivolumab (1 mg/kg) + ipilimumab (3 mg/kg) OR nivolumab (3 mg/kg) + ipilimumab (1 mg/kg).

c Only patients with high tumor mutation burden were included.

The IMpower133 trial evaluated the efficacy and safety of the anti-PD-L1 antibody conjugate atezolizumab in combination with carboplatin and etoposide in treatment-naive patients diagnosed with advanced SCLC.^60^^,^^79^ The investigators found that although the addition of atezolizumab to CHT significantly prolonged the PFS and OS of these patients (versus the placebo group; OS and PFS were 12.3 versus 10.3 months and 5.2 versus 4.3 months, respectively), the gain in survivals was relatively modest compared to those achieved in NSCLC.^79^ Nevertheless, results of this trial represent the first significant improvement in systemic therapy for untreated SCLC patients in the last 30 years. Accordingly, the IMpower133 study has prompted the US Food and Drug Administration (FDA) and the European Medicines Agency (EMA) to approve atezolizumab in this setting.^24^

Notably, similar results were observed in the CASPIAN study, where first-line durvalumab plus platinum-etoposide resulted in moderately improved OS and overall response rate (ORR) (versus a clinically relevant control group; OS rates were 13 versus 10.3 months, and ORRs were 79% versus 70%, respectively).^80^ Importantly, however, there were no significant differences in PFS (5.1 versus 5.4 months).^80^

Pembrolizumab is an anti-PD-1 antibody approved for metastatic SCLC patients with disease progression after platinum-based CHT and at least one other line of therapy.^81^ Results from the KEYNOTE-028 and KEYNOTE-158 studies revealed antitumor activity in a subset of patients with recurrent SCLC.^81^ The KEYNOTE-158 study enrolled patients regardless of their PD-L1 status, whereas the KEYNOTE-028 study only included patients with PD-L1-positive SCLCs. Based on the median OS of 9.7 (KEYNOTE-028) and 9.1 (KEYNOTE-158) months in patients treated with pembrolizumab, this agent constitutes a treatment option for recurrent SCLC as third- or subsequent-line therapy.^81^ The CheckMate 032 clinical trial evaluated the efficacy of nivolumab alone or in combination with ipilimumab.^28^^,^^82^ The patients eligible for the study received platinum-based CHT and at least one other agent, but they still showed a rapid disease progression. In addition, the study also demonstrated an improved cytotoxic activity for combined cytotoxic T lymphocyte-associated protein 4 (CTLA-4) (ipilimumab)- and PD-1 (nivolumab)-targeted antibodies.^82^ Of note, however, third-line therapy for metastatic SCLC patients with nivolumab monotherapy demonstrated a durable response in a subgroup of SCLC patients regardless of their PD-L1 status.^28^^,^^82^ Accordingly, in 2018, the FDA granted accelerated approval to nivolumab for patients with metastatic SCLC based on the results of the CheckMate 032 clinical trial.^28^

All in all, although some data suggest that SCLC should be more vulnerable to immunotherapy than NSCLC, the available clinical results are less compelling. Therefore, there is an unmet need to identify the determinants of ICI activity in SCLC that might differ from those in NSCLC and other solid tumors.^57^ Immune phenotypes as well as the NE and molecular subtypes may provide a better understanding of the underlying properties of SCLC patients. NE-high tumors with the immune desert phenotype are associated with decreased immune cell (CD8^+^ effector T cells primarily) infiltration compared to NE-low tumors.^36^^,^^38^ Expressions of indoleamine 2,3-dioxygenase (IDO) and poliovirus receptor (PVR), which are both important factors of the SCLC immune microenvironment, are also significantly lower in NE-high tumors (versus NE-low tumors).^38^^,^^83^, ^84^, ^85^ In addition, these tumors are associated with low T cell immunoglobulin and mucin domain-3 (TIM3) levels as well.^38^^,^^86^ Of note, TIM3 is a specific marker of lymphocyte exhaustion and is one of the most promising immune-checkpoint targets.^86^ Altogether, patients with NE-high tumors are less likely to respond to ICIs.^36^^,^^38^ Another possible explanation for the poor response rates to immunotherapy in SCLC may be that PD-L1 expression is much lower in SCLC compared to other solid tumors, and cancer cell PD-L1 expression seemingly does not correlate with ICI efficacy.^34^^,^^87^ Furthermore, due to the suppressed expression of major histocompatibility complex class I (MHC class I) in the tumor microenvironment, antigen presentation might also be defective.^88^ Thus, the alterations affecting the antigen presentation by MHC molecules may contribute to escape from T cell recognition and destruction.^34^^,^^87^^,^^88^ Expressions of HLA-A, HLA-B, HLA-C, and β_2_-microglobulin are also significantly lower in SCLC cell lines (versus NSCLC), and, consequently, these cell lines are less immunogenic when injected into immunocompetent mice.^34^^,^^88^, ^89^, ^90^ Finally, the specific clinical features of SCLC might also affect the beneficial use of ICIs.^74^ Most SCLC patients often require prolonged steroid therapy due to superior vena cava syndrome or brain metastases.^74^ Chronic steroids, however, are a known limitation for immunotherapy.^74^^,^^91^

Immunotherapy-related adverse events (irAEs) represent a major concern in SCLC.^74^ irAEs mostly include inflammatory or autoimmune complications with sometimes severe sequelae for patients.^74^^,^^92^ Although the exact pathophysiological mechanism of irAEs has not been fully uncovered in SCLC, it is suspected that the genetic predisposition and the latent (i.e., clinically asymptomatic) PNSs might play a key role.^92^ One possible mechanism is a T cell-mediated reaction to shared antigens that are expressed both in tumors and inflammatory lesions.^92^ Additionally, the development of autoantibodies might also contribute to the appearance of irAEs, just as the overactivation of innate and adaptive immune cells, which lead to increased cytokine secretion.^92^ Lastly, the role of the gut mucosal immune system and the gut microbiome in irAEs has also been intensively investigated.^93^^,^^94^

#### Targeted therapy

Several targeted agents are being tested for the treatment of SCLC (Table 2). However, to advance these treatment options, it is necessary to better understand the molecular alterations that have been already described in SCLCs.^5^^,^^6^^,^^108^

**Table 2:** Summary of clinical trials evaluating the safety and/or efficacy of targeted agents in advanced-stage SCLC patients

Study name	Study phase	Mechanism of action	Agent	Outcomes
ClinicalTrials.gov: NCT02289690^95^	I/II	PARP inhibitor	veliparib	safety: well tolerated
ClinicalTrials.gov: NCT01286987^96^	I	PARP inhibitor	talazoparib	safety: well tolerated
ClinicalTrials.gov: NCT01638546^97^	II	PARP inhibitor	veliparib	ORR: 39% versus 14% (p = 0.016)
				PFS:^a^ 3.8 versus 2.0 (p = 0.39)
				OS:^a^ 82 versus 7.0 (p = 0.50)
ECOG-ACRIN 2511^98^	II	PARP inhibitor	veliparib	ORR: 71.9% versus 65.6% (p = 0.57)
				PFS: 6.1 versus 5.5 (p = 0.06)
				OS: 10.3 versus 8.9 (p = 0.17)
TAHOE^99^	III	DLL3-targeted antibody drug conjugate	Rova-T	non-significant results
TRINITY^100^	II	DLL3-targeted antibody drug conjugate	Rova-T	non-significant results
ALTER 1202^101^	II	tyrosine kinase inhibitor	anlotinib	PFS: 4.1 versus 0.7 (p < 0.0001)
				OS: 7.3 versus 4.9 (p = 0.0210
ClinicalTrials.gov: NCT00154388^102^	II	tyrosine kinase inhibitor	imatinib	non-significant results
ClinicalTrials.gov: NCT01533181^103^	II	IGF-R1 inhibitor	linsitinib	non-significant results
ClinicalTrials.gov: NCT00869752^104^	I	IGF-R1 inhibitor	dalotuzumab	safety: well tolerated
ClinicalTrials.gov: NCT02038647^105^	II	AURKA inhibitor	alisertib	ORR: 22% versus 18% (p = 0.406)
				PFS: 3.32 versus 2.17 (p = 0.113)
				OS: 6.86 versus 5.58 (p = 0.714)
SALUTE^106^	II	VEGF inhibitor	bevacizumab	ORR: 58% versus 48%
				PFS: 5.5 versus 4.4
				OS: 9.4 versus 10.9
ClinicalTrials.gov: NCT02454972^107^	II	RNA polymerase II inhibitor	lurbinectedin	ORR: 35.2%
				PFS: 3.5
				OS: 9.3

PARP, poly(ADP-ribose) polymerase; DLL3, delta-like protein 3; IGF-R1, insulin-like growth factor 1 receptor; AURKA, aurora kinase A; VEGF, vascular endothelial growth factor.

a In months.

Compared to other lung cancer subtypes and normal lung epithelial cells, SCLC cells show a high PARP expression profile and are highly sensitive to PARP inhibitors.^98^^,^^109^ In addition, PARP inhibitors also enhance the effects of CHT and ionizing radiation both *in vivo* and *in vitro*.^109^^,^^110^ Therefore, the use of PARP inhibitors might be a promising targeted therapeutic approach in SCLC patients. The phase II clinical trial ECOG-ACRIN 2511 investigated the efficacy of the PARP inhibitor veliparib in untreated, advanced-stage SCLC patients.^98^ The investigators found that although patients treated with veliparib in combination with cisplatin and etoposide doublet had an improved OS compared to the control group (OSs were 10.3 versus 8.9 months), the results were not statistically significant.^98^ PARP inhibitor combinations might thus be attractive therapeutic approaches for SCLC patients, but predictive biomarkers are required to maximize their clinical efficacy. Of note, expression of the already mentioned SLFN11 strongly correlates with veliparib efficacy and may represent a potential biomarker for these patients.^97^

The delta-like ligand 3 (DLL3) is highly expressed in a subset of SCLCs with NE origin.^24^ The antibody-drug conjugate rovalpituzumab tesirine (Rova-T) binds DLL3 on these target-expressing cells to induce cell death.^24^ Rova-T is the first targeted therapeutic agent in SCLC to use DLL3 as a novel biomarker.^100^ The phase II TRINITY study investigated the efficacy and safety of Rova-T as a third-line agent in relapsed and refractory SCLC patients.^100^ The study results revealed modest anti-tumor activity of Rova-T, which also caused toxicity.^100^ The Rova-T development program with reference to the phase III MERU trial was considered ineffective in case of unselected patients and was terminated by the manufacturer.^111^ Notably, although the results show a lack of survival benefit, Rova-T may still be a promising therapeutic approach for properly selected SCLC patients.

The phase II ClinicalTrials.gov: NCT01045421 study tested the activity and safety of alisertib in patients with relapsed or refractory SCLC or with other tumors.^60^^,^^112^ Patients eligible for the study had to have undergone two or fewer previous cytotoxic regimens. The phase II clinical trial of alisertib as a monotherapy or in combination with other agents in multiple tumor types showed antitumor activity and provides a therapeutic strategy in relapsed SCLCs.^112^

#### Surgery

Several retrospective observational studies and cancer registries have previously provided encouraging long-term results in patients who underwent surgical resection for early stage SCLC.^113^, ^114^, ^115^, ^116^, ^117^ Unfortunately, current screening and diagnostic approaches clearly fail to identify early stage SCLC patients who might be eligible for surgery.^118^ Surgery is thus rarely performed in SCLC and approximately 80%–85% of SCLC patients are being diagnosed with extensive disease.^119^ Therefore, there is an urgent clinical need for novel biomarkers with the potential of enabling early SCLC diagnosis and of improved selection of surgical candidates. Recent data have demonstrated for example the predominance of the ASCL1 subtype in early SCLC lesions.^42^^,^^55^ Importantly, novel targeted drugs may also find their way to the armamentarium of SCLC therapies in the neoadjuvant setting. The development of such neoadjuvant approaches could even allow to offer surgery for SCLC patients with initially more advanced disease. Thus, a better understanding of the molecular subtypes might revolutionize the role of surgery in SCLC and raise the hope for better outcomes in the future.

### Targeted therapy with regard to NE and molecular subtypes

**Figure 3:**
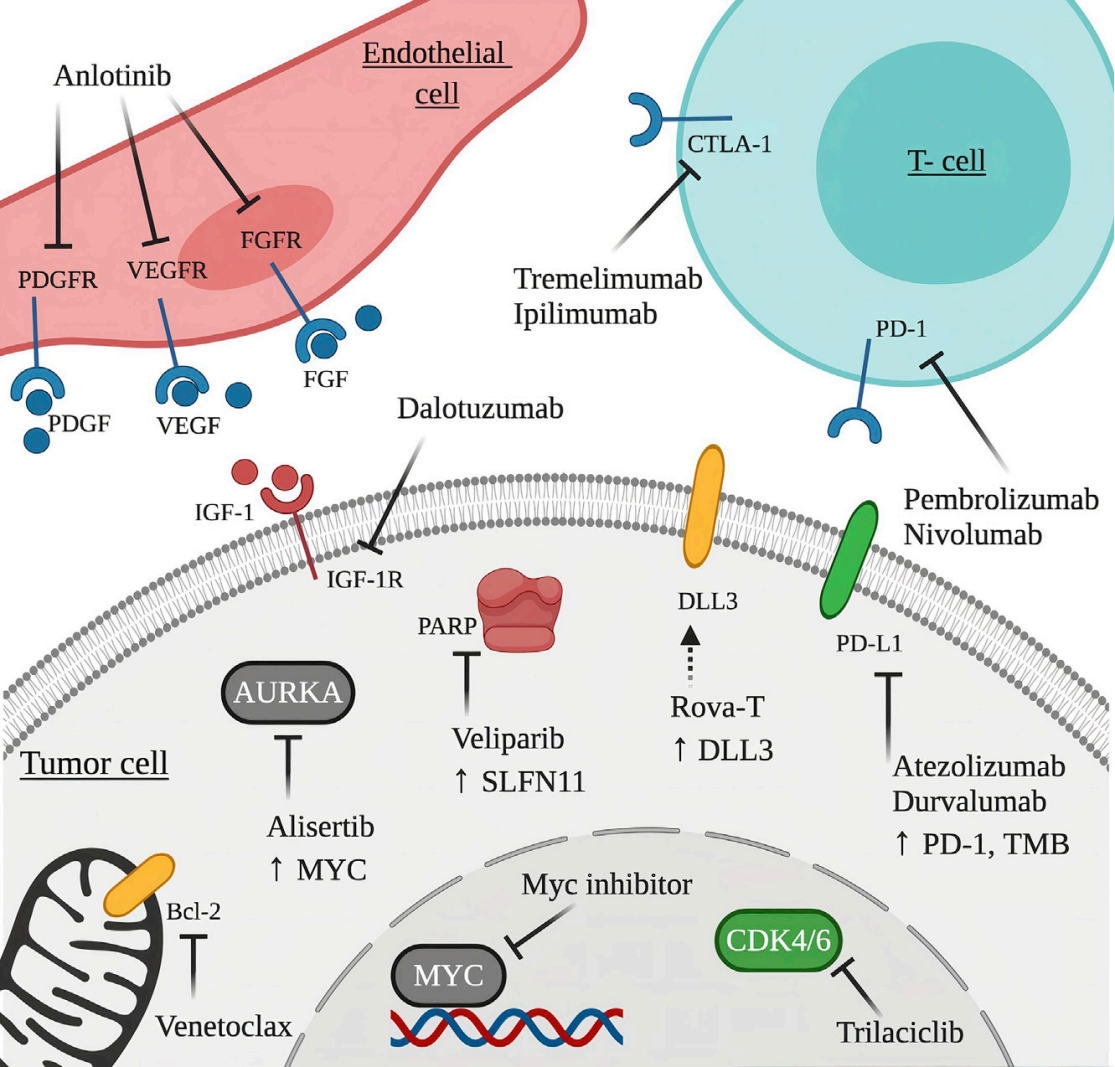
Potential novel therapeutic approaches in SCLC Subtype-specific potential therapeutic targets for SCLC-A, SCLC-N, SCLC-P, and SCLC-Y subtypes are highlighted in yellow (BCL2 and DLL3), gray (c-MYC and AURKA), red (PARP and IGF-1R), and green (CDK4/6 and PD-L1), respectively. The association between the aforementioned molecular subtypes and the potentially targetable molecules highlighted in blue (PDGFR, VEGFR, FGFR, CTLA-4 and PD-1), is currently unknown. The figure was created with BioRender.

Targeted therapies for SCLC have so far failed, and the success of immunotherapy in NSCLC has not been reflected in SCLC.^120^ One major reason for these relatively disappointing results lies behind the heterogeneity of SCLC.^33^^,^^47^ Unlike in NSCLC therapy and clinical studies, SCLC patients are still enrolled in clinical trials irrespective of their molecular background.^33^ Accordingly, identification of subtype-specific molecular profiles and clinically meaningful biomarkers may contribute to novel targeted strategies in SCLC (Figure 3).

#### SCLC-A

The SCLC-A subtype is anticipated to respond to DLL3-targeted antibody drug conjugates due to the direct transcriptional interaction of DLL3 with *ASCL1* in Notch^inactive^ tumor cells.^121^^,^^122^ Accordingly, treatment with the DLL3-targeted antibody drug conjugate Rova-T defines a subtype-specific therapy for SCLC-A.

*BCL2* is another direct transcriptional target of ASCL1, and the high expression levels are suggestive of potential therapeutic benefit from the BCL2 inhibitor venetoclax.

The histone demethylase LSD1 activity has been described to be dependent on the disruption of INSM1, which is linked to the NE subtypes SCLC-A and SCLC-N.^123^ Recent data also indicate that LSD1 inhibition leads to NOTCH1 activation, resulting in ASCL1 suppression in SCLC.^124^

In addition, the SCLC-A subtype is also associated with CREBBP inactivation and thus with increased sensitivity to histone deacetylase (HDAC) inhibitors (e.g., pracinostat).^125^

#### SCLC-N

The SCLC-N subtype is frequently associated with *MYC* amplification, which serves as a potential target for therapeutic agents.^56^ Increased AURKA activity and arginine biosynthesis are also characteristic features of this particular subtype.^55^ Accordingly, the combination therapy of both AURKA (e.g., alisertib) and c-MYC inhibitors is hypothesized to enhance the therapeutic efficacy.^33^^,^^126^ Additionally, the SCLC-N (i.e., “variant”) subtype exhibits a selective tropism for the oncolytic Seneca Valley virus (SVV),^127^ which infects and eliminates the NE cancer cells via lysis. Therefore, with appropriate biomarker-guided patient selection, the SVV oncolytic virus may have selective efficacy either as single agent therapy or in combination with immunotherapy.^33^^,^^127^^,^^128^ In this context, the NEUROD1-to-ASCL1 ratio may function as a predictive biomarker.^127^ Finally, based on recent *in vivo* studies, “NEUROD1-high” tumor cells are also suspected to be sensitive to arginine depletion caused by pegylated arginine deaminase (ADI-PEG 20), which leads to the inhibition of tumor cell growth.^33^^,^^54^

#### SCLC-P

Based on the results of CRISPR screens, the SCLC-P subtype possesses a unique vulnerability to IGF-1R deficiency.^46^ This leads to the hypothesis that IGF-1R inhibitors (e.g., dalotuzumab) may serve as potential specific therapeutic agents for these patients.^46^ PARP inhibitors (e.g., veliparib) are also suspected to be most effective in this molecular subtype, although, to the best of our knowledge, SLFN11 expression does not correlate with the subtype-specific transcriptional regulators.^129^ Lastly, we mention that the SCLC-P subtype might be sensitive to nucleoside analogues as well.^130^

#### SCLC-Y

PD-1 or PD-L1 expressions are not subtype-specific but may preferentially be linked to the SCLC-Y subtype since YAP1 has been shown to upregulate PD-L1 transcripts and induce an immunosuppressive tumor microenvironment.^131^^,^^132^ In addition, SCLC-Y tumor cells have higher expression levels of both CD38 and LAG-3 and, consequently, they show a higher likelihood to respond to ICIs.^131^ Based on gene expression and recent *in silico* results, the SCLC-Y subtype is also the most sensitive to mTOR, PLK, and potentially to CDK4/6 inhibitors.^45^^,^^133^

Metabolic pathways may also provide new targets for the treatment of SCLC. Recent data described an iron-dependent type of regulated necrosis called ferroptosis.^134^ Non-NE SCLCs were demonstrated to be selectively sensitive to induced ferroptosis.^134^ In contrast, high-NE SCLCs are resistant to induced ferroptosis, but respond exquisitely to thioredoxin (TRX) pathway inhibition.^134^ A combination of ferroptosis induction and TRX pathway inhibition may thus provide a treatment regimen for intratumoral NE/non-NE heterogeneous SCLC.^134^

### Open questions

#### What is the clinical relevance of intratumoral and intertumoral heterogeneity in SCLC?

The molecular characterization of SCLC is still of particular interest, although there have been major steps forward in identifying the exact pathogenesis and developing novel therapeutic approaches.^6^^,^^33^^,^^47^ SCLC is not a single entity as was thought before, but it displays heterogeneity in multiple ways. Significant intratumoral and intertumoral heterogeneity in SCLC was shown at the level of molecular diversity and NE differentiation. Bronchoscopic examination of the tumor may not be sufficient to determine the histological features and mutational landscape due to a possible presence of multiple subtypes within a tumor.^51^^,^^135^

#### Do NE and molecular subtypes correlate with different prognosis and clinical implications?

Genetic alterations lead to the development of SCLC subtypes and therapeutic resistance. The classification of NE (NE-high, NE-low, and non-NE) and molecular subtypes (ASCL1, NEUROD1, YAP1, and POU2F3) in recent years improved our understanding of SCLC. Recent clinical data demonstrate that ASCL1 overexpression might be a negative prognostic indicator in early stage resected SCLC patients.^122^ With regard to therapeutic implications, however, the SCLC-A subtype is suspected to be more chemosensitive compared to the SCLC-N subtype, since most SCLC-A cell lines are derived from treatment-naive patients whereas the SCLC-N cell lines mostly originate from post-treatment patients.^40^ As for the SCLC-Y subtype, YAP1 expression is linked with poor prognosis and decreased survival plus increased chemoresistance.^45^ These hypotheses are widely disputed, and the SCLC behavior in a clinical context is still not clarified.

#### What is the significance of biological plasticity between subtypes?

The presence of multiple subpopulations within a tumor and the biological plasticity between the subtypes might contribute to therapeutic outcomes. Recent preclinical studies suggest a possible hierarchy between subtypes, with SCLC-A being a necessary precursor of SCLC-N.^33^^,^^42^^,^^55^ This tumor evolution might greatly influence the response rates, as some tumor subpopulations may escape from therapy.^33^ Of note, however, the success of targeted therapies in cancer treatment is impaired by other mechanisms of resistance as well. Increased TMB potentially leads to a higher chance of developing drug resistance just as the amplification of the transcriptional regulator NFIB, driving tumor initiation, progression, and metastasis of SCLC.^5^

#### Do metabolic pathways in each subtype have therapeutic impact?

Beside the molecular background in SCLC, metabolic pathways in each particular subtype also influence the tumor behavior and therapeutic response. Ferroptosis and arginine depletion have recently been investigated to become targets for subtype-specific therapy.^54^^,^^134^ MYC-driven SCLC cells are dependent on different arginine-regulated pathways.^54^ Non-NE SCLCs are reported to be specifically sensitive to induced ferroptosis.^134^ Addressing selective cell death and metabolic pathways in SCLC subtypes may help to identify subtype-specific vulnerabilities for targeted therapies.^134^

#### Do SCLC subtypes display different metastatic potential and organotropism?

The NE pattern of LN or organ metastases might not reflect that of the primary tumor.^49^ Therefore, the resulting discordance between the primary tumor and metastases may result in the partial efficacy of therapeutic agents. Metastases of SCLC are observed to have a preference for certain organs. Very common sites for metastases comprise brain, bone, liver, and adrenal glands.^136^ Additionally, SCLC cells are suspected to arise from different cells of origin. The definition of the distinct precursor cells may reveal biomarkers, which help to understand the early events of tumorigenesis and predict the tumor evolution.^137^

### Conclusions

In contrast to NSCLC, where genotype-based targeted therapies have dramatically improved the treatment outcomes in patients with advanced stage disease, the therapy options in SCLC are still limited and the survival rates are dismal. No significant progress has been made in the systemic treatment of SCLC in the last three decades, mainly due to the high plasticity of SCLC and also to the non-selected patient groups in clinical trials. Recent research defined the heterogeneity in SCLC, but further exploration of the nature of SCLC subtypes is needed to interpret their similarities, diversities, and respective behavior. Defining the distinct gene expression profiles (*ASCL1*, *NEUROD1*, *POU2F3*, and *YAP1*) of SCLC patients will be fundamental to choose the most effective therapy. Because immunotherapies, biomarker-directed therapies, and chemotherapies operate on different targets and mechanisms, a combined or synergistic treatment may increase the therapeutic effects. Conclusively, the development of new drugs and a combination of different subtype-specific therapies are substantial to fight this deadly disease.
